# The complete chloroplast genome sequence of *Brachypodium distachyon*: sequence comparison and phylogenetic analysis of eight grass plastomes

**DOI:** 10.1186/1756-0500-1-61

**Published:** 2008-07-31

**Authors:** Esteban Bortiri, Devin Coleman-Derr, Gerard R Lazo, Olin D Anderson, Yong Q Gu

**Affiliations:** 1 Genomics and Gene Discovery Research Unit, USDA-Agriculture Research Service, Western Regional Research Center, 800 Buchanan Street, Albany, CA 94710, USA

## Abstract

**Background:**

Wheat, barley, and rye, of tribe Triticeae in the Poaceae, are among the most important crops worldwide but they present many challenges to genomics-aided crop improvement. *Brachypodium distachyon*, a close relative of those cereals has recently emerged as a model for grass functional genomics. Sequencing of the nuclear and organelle genomes of *Brachypodium *is one of the first steps towards making this species available as a tool for researchers interested in cereals biology.

**Findings:**

The chloroplast genome of *Brachypodium distachyon *was sequenced by a combinational approach using BAC end and shotgun sequences derived from a selected BAC containing the entire chloroplast genome. Comparative analysis indicated that the chloroplast genome is conserved in gene number and organization with respect to those of other cereals. However, several *Brachypodium *genes evolve at a faster rate than those in other grasses. Sequence analysis reveals that rice and wheat have a ~2.1 kb deletion in their plastid genomes and this deletion must have occurred independently in both species.

**Conclusion:**

We demonstrate that BAC libraries can be used to sequence plastid, and likely other organellar, genomes. As expected, the *Brachypodium *chloroplast genome is very similar to those of other sequenced grasses. The phylogenetic analyses and the pattern of insertions and deletions in the chloroplast genome confirmed that *Brachypodium *is a close relative of the tribe Triticeae. Nevertheless, we show that some large indels can arise multiple times and may confound phylogenetic reconstruction.

## Findings

Plastids are key organelles of green plants, carrying out functions like photosynthesis, starch storage, nitrogen and sulfate metabolism, and synthesis of chlorophyll, carotenoids, fatty acids and nucleic acids [1]. Plastids have multiple copies of a circular, double-stranded DNA chromosome, each with a set of approximately 110 genes highly conserved in sequence and organization [2].

In addition to their important biological roles, plastids have the potential to make a big impact on biotechnology. Plastid transformation, achieved via homologous recombination, is very advantageous compared to nuclear genome transformation mainly because it can generate high levels of gene expression and the recombinant DNA is more easily contained since chloroplasts are maternally inherited in most species of angiosperms [3].

The family Poaceae, with approximately 10,000 species, contains the world's most important crops. The tribe Triticeae, of subfamily Pooideae, includes species grown in temperate regions, some of which are of great economic importance; i.e., wheat, rye, triticale, and barley. Despite their contribution to human food supply, members of the Triticeae are not easily amenable to functional genomics aimed at crop improvement because of their large genome size and difficulty in transformation.

*Brachypodium distachyon*, a small grass in the Pooideae, has recently emerged as a new model species for functional genomics of temperate grasses. *Brachypodium *offers many advantages as a model grass; among them, its reduced stature, short life cycle, and small genome [4].

In the last few years a considerable effort has been made to develop genetic and molecular tools for *Brachypodium*, including ESTs [5], Bacterial Artificial Chromosome (BAC) libraries [6], cytological characterization of accessions [7-9], and techniques to perform rapid and efficient transformation [10,11]. Finally, sequencing of the *Brachypodium distachyon *genotype Bd21 has been initiated by the DOE Joint Genomics Institute and will soon be available to the public.

Here we report the sequencing of the chloroplast genome of the Bd21 genotype of *Brachypodium*, and perform a sequence analysis and phylogeny reconstruction with the completely sequenced chloroplast genomes from seven grass species. We compare the evolutionary dynamics of *Brachypodium *chloroplast genes with those of wheat, rice and maize, and discuss the significance of some indels in the framework of grass evolution.

### Sequencing of the Brachypodium chloroplast genome

**Figure 1:**
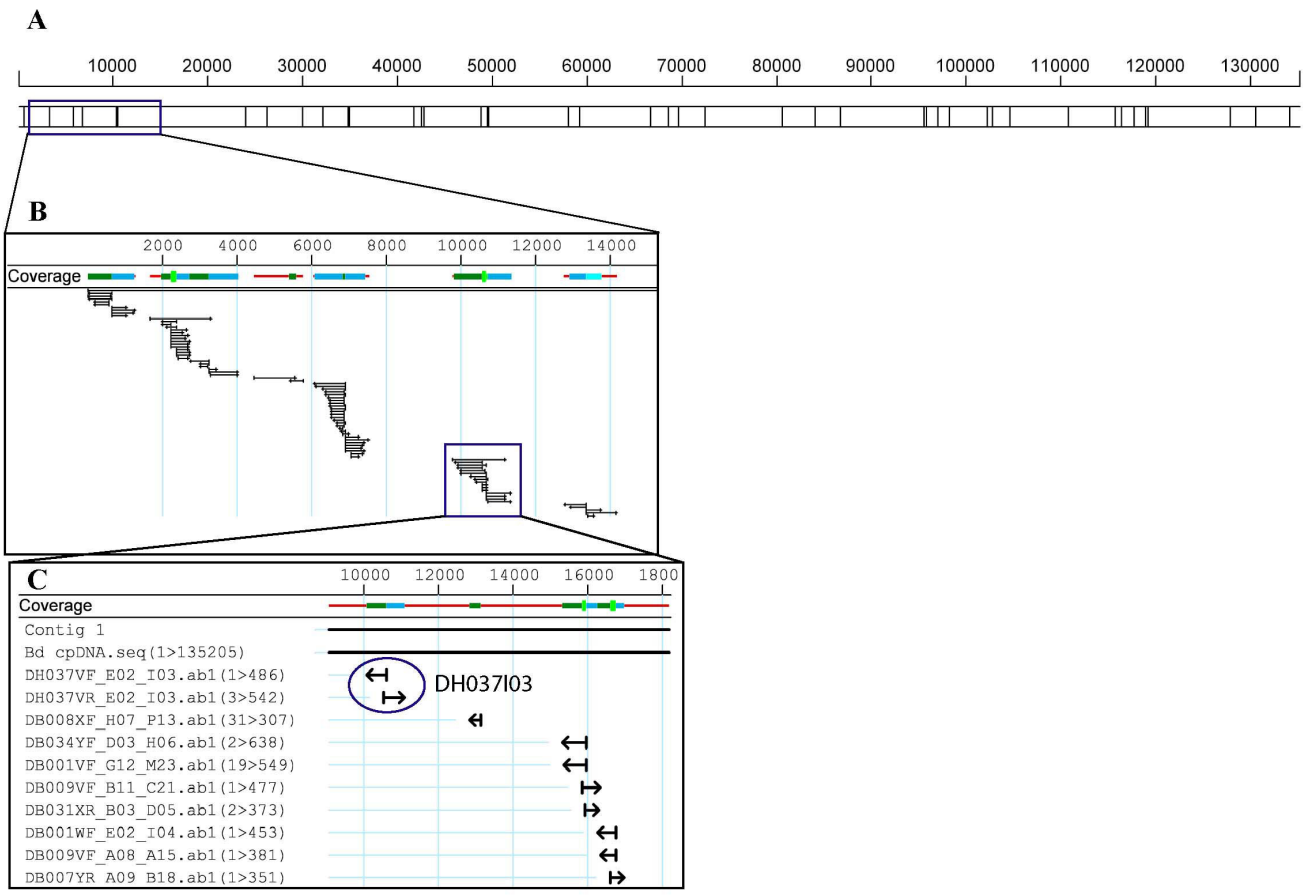
BAC end sequences (BES) coverage of the *Brachypodium distachyon *plastid chromosome. A: There are 43 HindIII sites in the *Brachypodium *and wheat plastid genomes, which explain the distribution of BAC end coverage. B: The *Brachypodium *BAC end sequences (BES) were assembled using the wheat chloroplast genome as a scaffold. C: clone DH037I03 contains the entire *Brachypodium *chloroplast genome indicated by its back-to-back BES (circled).

Sequencing of plastid genomes is usually done by isolation of chloroplasts followed by purification and amplification of plastid DNA for library construction. To sequence the chloroplast genome of *Brachypodium distachyon*, we took advantage of existing BAC libraries [12] and identified several chloroplast BACs from a database of BAC end sequences (BES). In our analysis, 1,725 BES matched wheat chloroplast queries. Clones generated from a single restriction of the chloroplast genome should contain the entire chloroplast genome and its two BES would assemble in the same region in opposite orientations. The two BES from BAC DH037I03 matched back-to-back the sequence of the wheat *psbC *gene (Fig. 1C). Overall, we identified over 30 BACs harboring the complete chloroplast genome, suggesting that this strategy is efficient in identifying full-length chloroplast genomes from genomic BAC libraries.

As expected, the chloroplast sequence assembled using the BES contained many gaps due to the distance between restriction sites (Fig. 1). To complete the *Brachypodium *chloroplast genome, a shotgun sequencing library of DH037I03 was constructed. The complete genome sequence was assembled using 1,725 BES, 410 sequences from the shotgun library, and 264 gap-filling sequences generated by primer walking. The sequence coverage of the entire chloroplast genome is 8.9×.

### Genome organization of Brachypodium chloroplast

**Figure 2:**
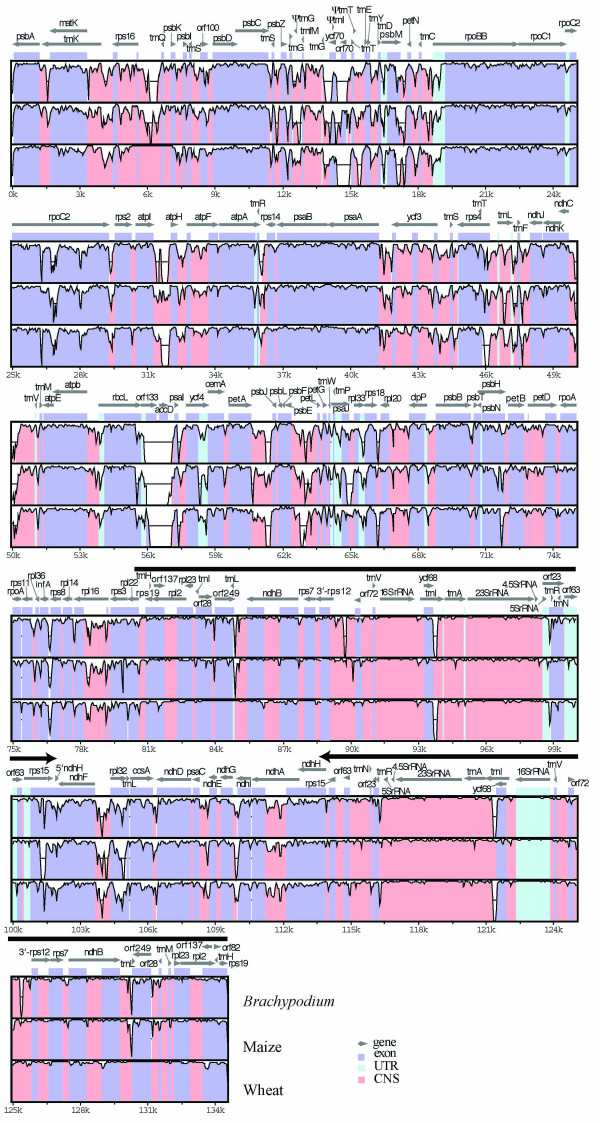
Alignment of grass chloroplast genomes. The sequence of rice chloroplast genome is compared to those of *Brachypodium *(top alignment), maize (middle), and wheat (bottom). Sequences were aligned in mVISTA [24] and the annotation shown above the alignment corresponds to the rice genome. Grey arrows above the alignment indicate genes and their orientation. Colors indicate location of exons, conserved non-coding sequences (CNS), and untranslated regions (UTRs). Ribosomal genes are colored as CNS. Thick black lines show the position of the IRs. Other grass genomes mentioned in the text have been omitted for the sake of simplicity.

The chloroplast genome of *Brachypodium distachyon *is 135,197 bp in length. The Inverted Repeats (IR) are 21,540 bp in length each, and the Large Single Copy (LSC) and Small Single Copy (SSC) regions are 79,446 bp and 12,668 bp long respectively. The *Brachypodium *chloroplast genome contains 118 unique genes, 18 of which are duplicated in the IRs, making a total of 136 genes of known function. In addition, there are 9 predicted open reading frames (ORFs) and 3 tRNA pseudogenes. With a few exceptions discussed below, the gene number and order are identical to other grass chloroplast genomes (Fig. 2).

### Grass chloroplast phylogeny based on complete chloroplast genomes

In a landmark article that included data from multiple sources, the Grass Phylogeny Working Group [13] examined relationships among grasses using a large and diverse assemblage of species. That study highlighted the existence of two major lineages, the BEP clade and the PACCAD clade, that together encompass the majority of grasses. The BEP clade includes the subfamilies Bambusoideae, Ehrhartoideae, and Pooideae. Rice belongs to subfamily Ehrhartoideae while wheat, barley, bentgrass, and *Brachypodium *are in the Pooideae. The PACCAD clade includes several subfamilies, among them the Panicoideae, a large group of mainly tropical and subtropical species, some of which are important crops worldwide, like maize, sugarcane, and sorghum.

**Figure 3:**
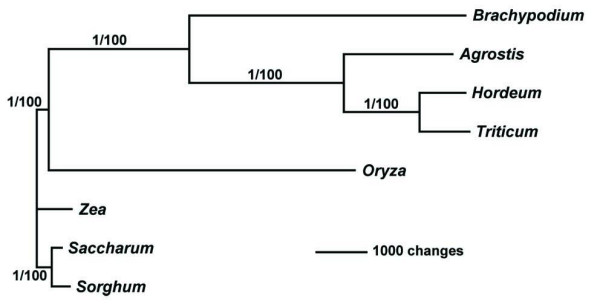
Complete chloroplast genome phylogeny of the grasses. The phylogram was obtained from an exhaustive parsimony search and was the same to a topology obtained from a Bayesian analysis. The tree was rooted making maize, sugarcane, and sorghum the outgroup. Support for the nodes is shown as posterior probability after 1000000 generations and bootstrap values from 1000 repetitions. The GenBank accesions used for the analyses are X15901 (rice), EU325680 (*Brachypodium*), EF115543 (bentgrass), EF115541 (barley), X86563 (maize), AP006714 (sugarcane), EF115542 (sorghum), and AB042240 (wheat). The sequences were aligned and visualized using mVISTA [25]. MrBayes [14] and PAUP* [24] were used to analyze the data.

So far, all phylogeny reconstructions of the Poaceae have used selected genes or partial regions as data. However, with sequenced chloroplast genomes of several species in this family and the computer power to align them, it is possible for the first time to perform whole chloroplast genome phylogenic analyses. To examine if the genome-wide phylogenic analysis is consistent with those based on selected genes, we employed Bayesian [14] and Maximum Parsimony [15] methods to reconstruct a grass phylogeny using whole chloroplast sequences. Both Bayesian and Maximum Parsimony estimates produced the same topology with maximum node support (Fig. 3). The topology shown on Fig. 3 contained 99% of the Bayesian credible trees and the tree is in agreement with the results obtained with a larger group of species [13]. The phylogram also shows that branches in the BEP clade are much longer than those in the PACCAD clade. A similar result was found by Saski et al. [16] in a phylogenetic study using 61 protein-coding genes, indicating that the rates of evolution are higher in the BEP clade compared to the PACCAD species sampled here. However, it is possible that these slower rates do not extend to other species of the PACCAD clade, since maize, sorghum, and sugarcane are closely related, with all three belonging to subfamily Panicoideae.

### Evolution of Brachypodium chloroplast genes

For a given protein-coding gene, the proportion of substitutions that do not cause a change in the amino acid sequence (synonymous) to those that do (nonsynonymous) is a commonly used estimator of the evolutionary dynamics operating on that gene [15]. To find out if *Brachypodium *plastid genes show the same evolutionary dynamics as other grasses we calculated the ratio of nonsynonymous to synonymous substitution rates for *Brachypodium *chloroplast genes using tobacco as an outgroup.

We found that the nonsynonymous/synonymous ratios for *Brachypodium *chloroplast genes are similar to those of rice, maize and wheat, with photosynthetic genes having the lowest ratio (Table 1), in agreement with previous findings [17]. Within the NADH class, *ndh*B and *rps*12 have very low rates of both kinds of substitutions compared to other genes in the same class, a result explained by their position, in the IRs and most likely due to the dynamics of the IRs' evolution rather than to evolutionary constrains on *ndh*B and *rps*12.

**Table 1:** Substitution rates in grasses. Chloroplast genes are divided into seven groups according to the function of their product. For groups of more than one gene the top row gives the mean substitution rate, and the second and third rows show the genes, within that group, with the maximum and minimum rates respectively. ENV: Envelope membrane. MAT: *maturase*K. NADH: NADH genes. PS: Photosynthetic genes. RP: ribosomal genes. RNPol: RNA polymerase genes. B: *Brachypodium*. W: wheat. R: rice. M: maize.

**GENE GROUP**	**NO. OF GENES**	**SYNONYMOUS SUBSTITUTIONS^1^**				**NONSYNONYMOUS SUBSTITUTIONS^1^**				**NNONSYNONYMOUS/SYNONYMOUS RATIO**			
		
		**B**	**W**	**R**	**M**	**B**	**W**	**R**	**M**	**B**	**W**	**R**	**M**
**RNPol**	4	0.371	0.359	0.358	0.36	0.131	0.126	0.131	0.128	0.35	0.35	0.37	0.35
Max		*rpo*C1 0.452	*rpo*C1 0.423	*rpo*C1 0.428	*rpo*C1 0.42	*rpo*C2 0.172	*rpo*C2 0.162	*rpo*C2 0.166	*rpo*C2 0.159	*rpo*C2 0.6	*rpo*C2 0.57	*rpo*C2 0.61	*rpo*C2 0.54
Min		*rpo*C2 0.289	*rpo*C2 0.286	*rpo*C2 0.273	*rpo*C2 0.296	*rpo*B 0.088	*rpo*B 0.084	*rpo*B 0.087	*rpo*B 0.089	*rpo*C1 0.24	*rpo*B 0.24	*rpo*C1 0.25	*rpo*C1 0.25
**ENV**	1	0.46	0.454	0.422	0.44	0.2	0.193	0.2	0.188	0.44	0.43	0.47	0.43
**MAT**	1	0.448	0.47	0.466	0.45	0.225	0.209	0.212	0.213	0.5	0.44	0.46	0.47
**NADH**	11	0.362	0.354	0.358	0.36	0.092	0.092	0.091	0.09	0.25	0.26	0.25	0.25
Max		*ndh*H 0.463	*ndh*H 0.461	*ndh*H 0.445	*ndH*A 0.448	*ndh*F 0.188	*ndh*F 0.18	*ndh*F 0.181	*ndh*F 0.172	*ndh*F 0.55	*ndh*F 0.53	*ndh*F 0.51	*ndh*F 0.49
Min		*ndh*B 0.062	*ndh*B 0.076	*ndh*B 0.059	*ndh*B 0.065	*ndh*B 0.028	*ndh*B 0.04	*ndh*B 0.029	*ndh*B 0.025	*ndh*C 0.125	*ndh*H 0.14	*ndh*H 0.13	*ndh*H 0.15
**PS**	32	0.370	0.362	0.353	0.36	0.039	0.041	0.039	0.039	0.11	0.12	0.11	0.11
Max		*psa*C 0.554	*psa*C 0.571	*psa*C 0.519	*psa*C 0.485	*atp*E 0.158	*atp*E 0.158	*atp*E 0.15	*atp*E 0.147	*atp*E 0.42	*atp*F 0.42	*atp*F 0.47	*atp*F 0.53
Min		*psb*L 0.2	*psb*L 0.16	*psb*L 0.16	*psb*L 0.16	*pet*G, *psb*I 0	*pet*G, *psb*I 0	*pet*G *psb*M *psb*T 0	*pet*G, *psb*F *psb*I *psb*T 0	*pet*D 1.24 × 10^-2^	*pet*B 9.52 × 10^-3^	*pet*D 1.7 × 10^-2^	*pet*D 6.83 × 10^-3^
**PR**	1	0.442	0.412	0.426	0.427	0.161	0.161	0.168	0.161	0.36	0.39	0.39	0.38
**RP**	22	0.312	0.297	0.302	0.287	0.121	0.121	0.127	0.121	0.39	0.41	0.42	0.42
Max		*rps*8 0.489	*rps*8 0.469	*rps*8 0.48	*rps*8 0.47	*rpl*22 0.251	*rpl*22 0.248	*rpl*22 0.256	*rpl*22 0.256	*rpl*2 1.1	*rpl*2 0.98	*rpl*2 1.18	*rpl*2 1.18
Min		*rps*12 0.05	*rps*12 0.045	*rps*12 0.05	*rps*12 0.056	*rps*12 0.028	*rps*12 0.028	*rps*12 0.035	*rps*12 0.026	*rps*14 0.2	rpl16 0.21	*rps*14 0.2	*rps*14 0.18

^1 ^Rates were calculated using the method of Nei and Gojobori [27] using tobacco as an outgroup to *Brachypodium*, wheat, rice, and maize.

The rate of evolution of a particular gene; i.e., the estimated number of substitutions per site, can vary among different organisms for reasons like rapid gene duplication that creates opportunity for sequence divergence, different generation time, and various DNA repair mechanisms [15]. We conducted a relative rate test [18] for all *Brachypodium *chloroplast genes with known function against their orthologs in maize, wheat, and rice and found that most *Brachypodium *genes evolve at similar rates to those of wheat, rice, and maize. However, there are unequal rates of evolution (at *P *= 0.05) in 15 genes and 17 cases of species comparisons, and *Brachypodium *genes evolved at a faster rate in 14 out of those 17 comparisons (Table 2).

**Table 2:** Summarized results of Tajima's [18] test of relative evolution of *Brachypodium *chloroplast genes compared with those of wheat, rice, and maize. The *P *value of genes that evolve at significantly different rates in *Brachypodium *is shown for each gene and species comparison. When *P *< 0.05, indicating that rates are significantly different, the species with the highest rate of evolution is shown in parenthesis. B: *Brachypodium*, W: wheat, R: rice, M: maize.

Gene	Species pair comparison		
	B vs W	B vs R	B vs. M
*pet*A	0.04 (W)	1	0.87
*pet*B	0.23	0.79	0.04 (B)
*psb*B	0.67	0.04 (B)	0.17
*psb*H	0.37	0.02 (B)	1
*rps*11	0.011(B)	0.089	0.23
*rps*14	0.48	0.11	0.03 (B)
*rps*4	0.47	0.039 (B)	0.22
*rpl*2	0.00042 (W)	0.44	0.033 (B)
*rpl*20	0.011 (B)	0.64	0.18
*ndh*B	0.00023 (W)	0.66	0.47
*ndh*I	0.18	0.003 (B)	0.02 (B)
*trn*I	0.046 (B)	0.059	1
*rpo*C1	0.017 (B)	0.13	0.088
*rpo*C2	0.0081 (B)	0.055	0.062
*ycf*9	0.025 (B)	0.71	0.76

### Sequence comparison among grass chloroplast genomes

The structure and gene number of the chloroplast genome is very similar among land plants, although the Poaceae have three large inversions compared to the canonical plastid genome usually represented by the tobacco chloroplast genome [19]. This conservation of overall structure in the chloroplast genomes of grasses allowed us to align the chloroplast genome sequences of eight grass species at the genome-wide level.

Comparison of the sequences of eight chloroplast genomes (only rice, *Brachypodium*, wheat, and maize are represented on Fig. 2) reveals several regions of high sequence length polymorphism, as well as shared deletions and insertions. The IRs show lower sequence divergence among grasses than the single-copy region (Fig. 1), a result previously reported by other authors [20]. The region between *rbc*L and *psa*I (at position ~54 kb, Fig. 2) is one of the most polymorphic chloroplast loci in grasses. In rice, this region is 1532 bp long and contains ORF133 and the *acc*D gene, but it is much shorter in other grasses. In *Brachypodium*, both ORF133 and *acc*D are missing, and the entire *rbc*L-*psa*I spacer region, containing only the *rbc*L 3'UTR and *psa*I promoter sequences, is reduced to 296 bp long.

As expected from its phylogenetic placement, *Brachypodium *shares several indels with barley, wheat, and bentgrass, all of which are in subfamily Pooideae, including a 410 bp deletion in ORF70 (~14.5 kb, Fig. 2) and the duplication of a 5' portion of *ndh*H IRb (~102 K in Fig. 2) that is also shared with rice [16,21]. The size of this duplication is variable, ranging from 238 bp in rice to 311 bp in *Brachypodium*. Insertions in *rpo*C2 (~25 K, Fig. 2) have been described and used previously in phylogenetic analyses [[13], and references therein] and will not be discussed here.

### Rice and wheat have identical and independently derived deletions

**Figure 4:**
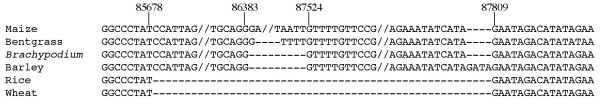
Deletions in the IR region. Rice and wheat have an identical 2.1 kb deletion in both IRs (indicated by the dashes). *Brachypodium*, bentgrass, and barley have a 1.14 kb deletion in the same region. The sequences flanking the deletions are shown. The positions shown on top of the alignment correspond to the maize sequence. Two slashes indicate that the sequence continues but is not shown here.

Despite the overall sequence conservation of IRs, the region between *ndh*B and *trn*I (~84 K and ~131 in Fig. 2) appears to be a hot spot for large indels. Previously, Ogihara et al [21] described a 2,131 bp deletion in wheat and rice with respect to maize. This deletion is located between ORF249 and ORF28 (~84 K and ~131 K, Fig. 2). Because rice is more closely related to wheat than to maize, the authors concluded that the deletion was present in the common ancestor of rice and wheat. However, this deletion is present only in rice and wheat, which are not sister species (Fig. 3), whereas in *Brachypodium*, barley, and bentgrass there is a smaller deletion of about 1,141 bp (Fig. 4).

To confirm that the 2,131-bp deletion in rice and wheat was not an artifact of the alignment or missing sequence, we used the *Brachypodium *sequence missing in wheat and rice and blasted it against grass sequence databases. We recovered sequences from many grasses except wheat and rice, confirming the presence of the deletion in their genomes. In addition, we searched the GenBank angiosperm databases with the maize sequence corresponding to the deleted wheat and rice region and found that the region is present in species representing diverse lineages of flowering plants, including the monocot *Dioscorea*, the early-diverging angiosperms *Amborella *and *Nymphaea*, and several core eudicots (data not shown). Therefore, we concluded that the 2,131-bp deletions in the wheat and rice chloroplast genomes are derived characters that arose independently in those species.

The 2,131-bp deletions in rice and wheat are identical in both IRs and the sequences bordering them align unambiguously with those of other grasses (Fig. 4). In addition, the lack of direct short repeats in sequences indicates that recombination *via *short repeats is not the way by which they arose. Thus, despite the fact that deletions of varying lengths in the *ndh*B-*trn*I region seem to be common in the BEP clade, the mechanism underlying these specific deletions remains unclear. In tobacco, nucleotide mutations in plastid coding sequences are quickly eliminated by gene conversion, a process facilitated by the polyploid nature of the plastid genome [22]. Whatever the mechanism is that generates deletions in the *trn*I-*ndh*B region in species of the BEP clade, their multiple occurrences suggests that they may provide a selective advantage to those species in order to overcome gene conversion and become fixed in the population.

## Competing interests

The authors declare that they have no competing interests.

## Authors' contributions

EB did the sequence alignment and comparison, the phylogenetic analyses, the relative tests of evolution, and drafted the manuscript. DC-D performed the BAC end sequence searches, BAC shotgun library construction and sequencing, sequence assemblage, and substitution rates analyses. GRL wrote the algorithm to search BES with wheat queries and assembled BES on the genome. YGU designed and coordinated the study. Both OA and YQG supervised the work and collaborated in the manuscript preparation. All authors have read and approved the final version of the manuscript.

## References

[B1] Staehelin LA, Newcomb EH, Buchanan BB, Gruissem W, Jones RL (2000). Membrane structures and membranous organelles. Biochemistry and Molecular Biology of Plants.

[B2] Palmer JD, Hermann RG (1991). Plastid chromosomes: structure and evolution. The molecular biology of plastids Cell culture and somatic cell genetics of plants.

[B3] Bock R (2007). Plastid biotechnology: prospects for herbicide and insect resistance, metabolic engineering and molecular farming. Current Opinion in Biotechnology.

[B4] Garvin DF, Gu YQ, Hasterok R, Hazen SP, Jenkins G, Mockler TC, Mur LAJ, Vogel J (2008). Development of genetic and genomic research resources for Brachypodium distachyon, a new model system for grass crop research. The Plant Genome [A Supplement to Crop Science].

[B5] Vogel J, Gu YQ, Twigg P, Lazo G, Laudencia-Chingcuanco D, Hayden DM, Donze TJ, Vivian LA, Stamova B, Coleman-Derr D (2006). EST sequencing and phylogenetic analysis of the model grass Brachypodium distachyon. Theoretical and Applied Genetics.

[B6] Huo N, Gu YQ, Lazo G, Vogel J, Coleman-Derr D, Luo M-C, Thilmony R, Garvin DF, Anderson OD (2006). Construction and characterization of two BAC libraries from Brachypodium distachyon, a new model for grass genomics. Genome.

[B7] Hasterok R, Draper J, Jenkins G (2004). Laying the cytotaxonomic foundations of a new model grass, Brachypodium distachyon (L.) Beauv. Chromosome Research.

[B8] Jenkins G, Hasterok R (2007). BAC 'landing' on chromosomes of Brachypodium distachyon for comparative genome alignment. Nature Protocols.

[B9] Hasterok R, Marasek A, Donnison IS, Armstead I, Thomas A, King IP, Wolny E, Idziak D, Draper J, Jenkins G (2006). Alignment of the genomes of Brachypodium distachyon and temperate cereals and grasses using bacterial artificial chromosome landing with fluorescence in situ hybridization. Genetics.

[B10] Vogel J, Garvin DF, Leong O, Hayden DM (2005). Agrobacterium-mediated transformation and inbred line development in the model grass Brachypodium distachyon. Plant Cell, Tissue and Organ Culture.

[B11] Christiansen P, Andersen CH, Didion T, Folling M, Nielsen KK (2005). A rapid and efficient transformation protocol for the grass Brachypodium distachyon. Plant Cell Reports.

[B12] Huo N, Lazo G, Vogel J, You FM, Ma Y, Hayden DM, Coleman-Derr D, Hill TA, Dvorak J, Anderson OD (2007). The nuclear genome of Brachypodium distachyon: analysis of BAC end sequences. Functional and Integrative Genomics.

[B13] GPWG (2001). Phylogeny and subfamilial classification of the grasses (Poaceae). Annals of the Missouri Botanical Garden.

[B14] Huelsenbeck JP, Ronquist F (2001). MRBAYES: Bayesian inference of phylogeny. Bioinformatics.

[B15] Nei M, Kumar S (2000). Molecular Evolution and Phylogenetics.

[B16] Saski C, Lee S-B, Fjellheim S, Guda C, Jansen R, Luo H, Tomkins J, Rognli OA, Daniell H, Clarke JL (2007). Complete chloroplast genome sequences of Hordeum vulgare, Sorghum bicolor, and Agrostis stolonifera, and comparative analyses with other grass genomes. Theoretical and Applied Genetics.

[B17] Matsuoka Y, Yamazaki Y, Ogihara Y, Tsunewaki K (2002). Whole chloroplast genome comparison of rice, maize, and wheat: implications for chloroplast gene diversification and phylogeny of cereals. Molecular Biology and Evolution.

[B18] Tajima F (1993). Simple methods for testing the evolutionary clock hypothesis. Genetics.

[B19] Doyle JJ, Davis JI, Soreng RJ, Garvin DF, Anderson MJ (1992). Chloroplast DNA inversions and the origin of the grass family (Poaceae). Proceedings of the National Academy of Sciences.

[B20] Yamane K, Yano K, Kawahara T (2006). Pattern and rate of indel evolution inferred from whole chloroplast intergenic regions in sugarcane, maize, and rice. DNA Research.

[B21] Ogihara Y, Isono K, Kojima T, Endo A, Hanaoka M, Shiina T, Terachi T, Utsugi S, Murata M, Mori N (2002). Structural features of a wheat plastome as revealed by complete sequencing of chloroplast DNA. Molecular Genetics and Genomics.

[B22] Khakhlova O, Bock R (2006). Elimination of deleterious mutations in plastid genomes by gene conversion. The Plant Journal.

[B23] Mayor C, Brudno M, Schwartz JR, Poliakov A, Rubin EM, Frazer KA, Pachter LS, Dubchak I (2000). VISTA: Visualizing global DNA sequence alignments of arbitrary length. Bioinformatics.

[B24] Swofford DL (2003). PAUP*. Phylogenetic analysis using parsimony (*and other methods), version 4.

[B25] Nei M, Gojobori T (1986). Simple methods for estimating the numbers of synonymous and nonsynonymous nucleotide substitutions. Molecular Biology and Evolution.

